# Quorum-sensing, intra- and inter-species competition in the staphylococci

**DOI:** 10.1099/mic.0.001381

**Published:** 2023-08-14

**Authors:** Paul Williams, Phil Hill, Boyan Bonev, Weng C. Chan

**Affiliations:** ^1^​ Biodiscovery Institute and School of Life Sciences, University of Nottingham, University Park, Nottingham, NG7 2RD, UK; ^2^​ School of Biosciences, Sutton Bonington Campus, University of Nottingham, Loughborough, LE12 5RD, UK; ^3^​ School of Pharmacy, Biodiscovery Institute, University of Nottingham, University Park, Nottingham, NG7 2RD, UK

**Keywords:** *agr*, autoinducing peptides, inter-bacterial competition, quorum sensing, staphylococci, *Staphylococcus aureus*

## Abstract

In Gram-positive bacteria such as *Staphylococcus aureus* and the coagulase-negative staphylococci (CoNS), the accessory gene regulator (*agr*) is a highly conserved but polymorphic quorum-sensing system involved in colonization, virulence and biofilm development. Signalling via *agr* depends on the interaction of an autoinducing peptide (AIP) with AgrC, a transmembrane sensor kinase that, once phosphorylated activates the response regulator AgrA. This in turn autoinduces AIP biosynthesis and drives target gene expression directly via AgrA or via the post-transcriptional regulator, RNAIII. In this review we describe the molecular mechanisms underlying the *agr*-mediated generation of, and response to, AIPs and the molecular basis of AIP-dependent activation and inhibition of AgrC. How the environment impacts on *agr* functionality is considered and the consequences of *agr* dysfunction for infection explored. We also discuss the concept of AIP-driven competitive interference between *S. aureus* and the CoNS and its anti-infective potential.

## Introduction

Coagulase-negative staphylococci (CoNS) such as *Staphylococcus epidermidis* and *Staphylococcus hominis* are primarily skin commensals while the coagulase-positive *Staphylococcus aureus* is not only a commensal colonizing human nares and skin but also a major opportunistic pathogen [1, 2]. *S. aureus* infections can be minor or invasive and life-threatening [3]. They may be acute or chronic ranging from skin, soft tissue and medical device-related to bacteraemia, endocarditis, osteomyelitis, food poisoning, septic arthritis, scalded skin and toxic shock syndromes [3]. *S. aureus* is also on the WHO ‘ESKAPE’ list of multi-antibiotic resistant priority pathogens given that treatment of *S. aureus* infections has been compounded by the emergence of multi-antibiotic-resistant strains [4]. These include vancomycin-resistant (VRSA and VISA) and methicillin (MRSA)-resistant isolates, which can be further sub-divided into hospital-acquired MRSA (HA-MRSA) and community-acquired (CA-MRSA) strains. *S. aureus* has often been reported as a co-infecting microbe in polymicrobial infections where it may be co-operative or competitive [5]. In the context of difficult-to-eradicate infections such as non-healing diabetic foot ulcers and in the lungs of individuals with cystic fibrosis (CF), co-infections of *S. aureus* with *P. aeruginosa* are indicative of much poorer clinical outcomes than in those infected with either species alone [6].

As both commensal and pathogen, *S. aureus* is capable of rapidly responding and adapting to fluctuating host and inanimate environments and switching between colonization and pathogenic modes. Such adaptation depends on local extracellular signals that include oxygen availability, temperature, pH, nutrient limitation, bacterial cell population density, other co-localizing microbes, antimicrobials and is associated with specific host tissue interactions, implanted medical devices, abiotic surfaces encountered in households or hospitals as well as particulate air pollution [7–9].

**Fig. 1.**
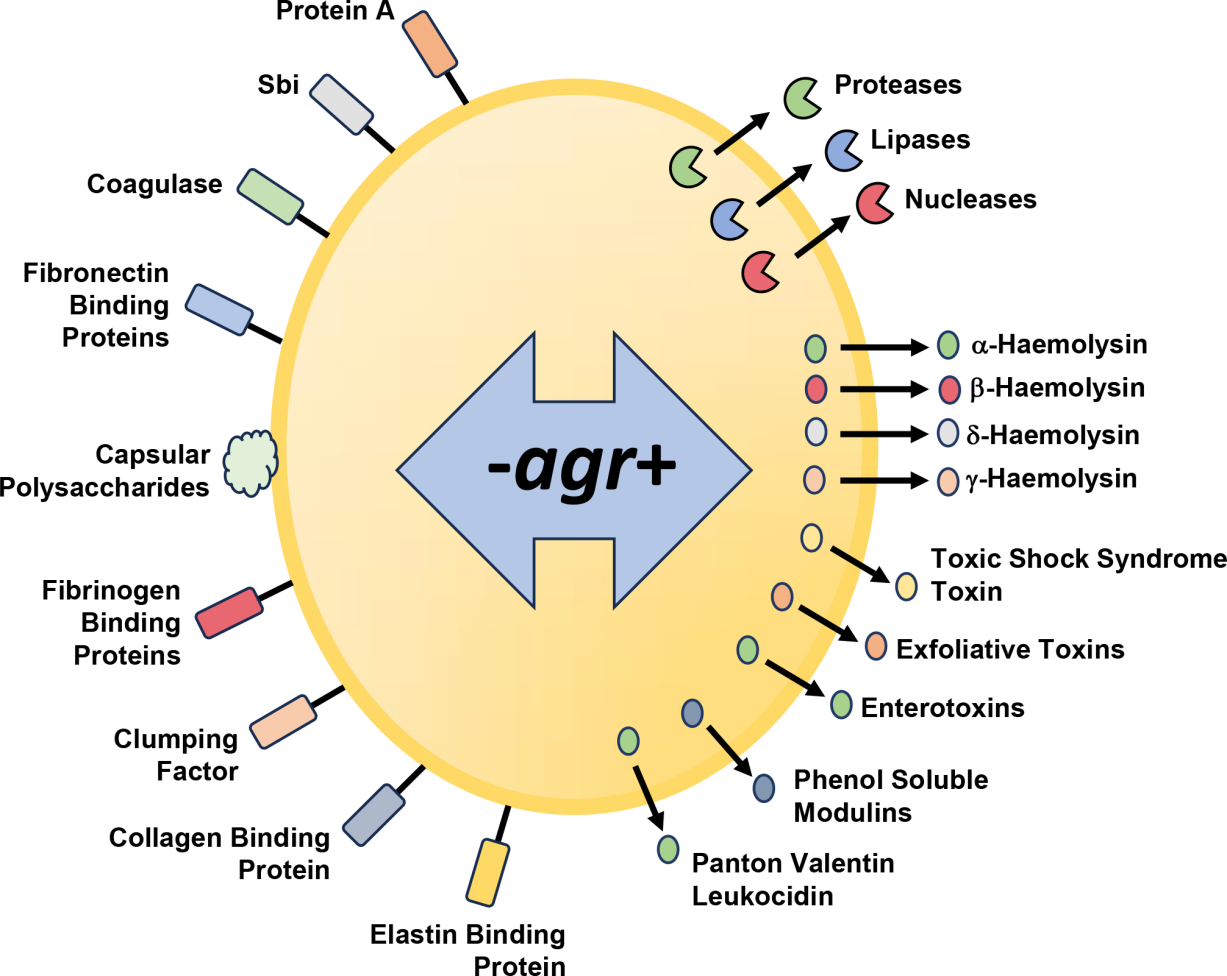
The *agr* QS system in *S. aureus* negatively (-) regulates the production of capsular polysaccharides and multiple cell-wall proteins involved in host protein interactions including immunoglobulin, fibronectin and fibrinogen binding proteins. *agr* positively (+) regulates the expression of diverse virulence factor genes including those coding for tissue degrading exoenzymes, haemolysins, enterotoxins, exfoliative toxins leukocidins and phenol soluble modulins.

The versatility of *S. aureus* as a pathogen revolves around diverse cell-wall colonization factors, immune modulating agents and secreted exoproducts [3], many of which are controlled via quorum sensing (QS), a mechanism for synchronizing gene expression via self-generated diffusible signal molecules in a population-dependent manner [10]. In Gram-positive pathogens including the staphylococci, clostridia, enterococci and listeria, QS is mediated by the accessory gene regulator (*agr*) system [11, 12]. In *Staphylococcus aureus *, *agr* reciprocally regulates multiple cell-wall proteins (e.g. immunoglobulin and fibronectin-binding proteins) and exotoxins (e.g. haemolysins, enterotoxins, leucocidins, toxic shock syndrome toxin, exoenzymes (nucleases, proteases, lipases) and the phenol soluble modulins (PSMs), a family of short amphipathic peptides with cytolytic activity similar to δ-toxin [3] (Fig. 1). Staphylococcal surface proteins promote adherence to host tissues and aid immune evasion while exotoxins cause tissue damage and many function as super-antigens promoting the onset of shock-like syndromes [3, 13]. The *S. aureus agr* system is involved in endosomal escape, intracellular survival and replication [14–16]. With respect to biofilm development, *agr* contributes to initial attachment, structuring and dispersal [17].

In acute animal models of skin, soft tissue, respiratory, arthritis and bone, *S. aureus agr* mutants are attenuated, highlighting a key role for QS-dependent regulation of virulence determinants at these infection sites [18]. Paradoxically, *agr* is required for skin colonization [19]. However, a functional *agr* system is dispensable for chronic, biofilm-related infections associated with, for example, implanted medical devices and cystic fibrosis [18]. Furthermore, allelic variation in *agr* genes contributes to intra- and inter-staphylococcal competition since the cognate *agr* signal molecule of one staphylococcal *agr* variant may inhibit *agr* in a strain possessing a different *agr* variant [20–22]. In this review we outline the intricate molecular mechanisms underlying the *agr*-mediated generation of, and response to, autoinducing peptide (AIP) signal molecules focusing primarily on *S. aureus *. We describe the specifics of AIP-activation and inhibition of the sensor kinase AgrC, how the environment impacts on *agr* functionality and explore the consequences of *agr* dysfunction for infection. We also discuss the concept of AIP-mediated competitive interference between *S. aureus* and the CoNS and its therapeutic potential for suppressing *S. aureus* skin disease and other infections.

### The *S. aureus agr* system

**Fig. 2.**
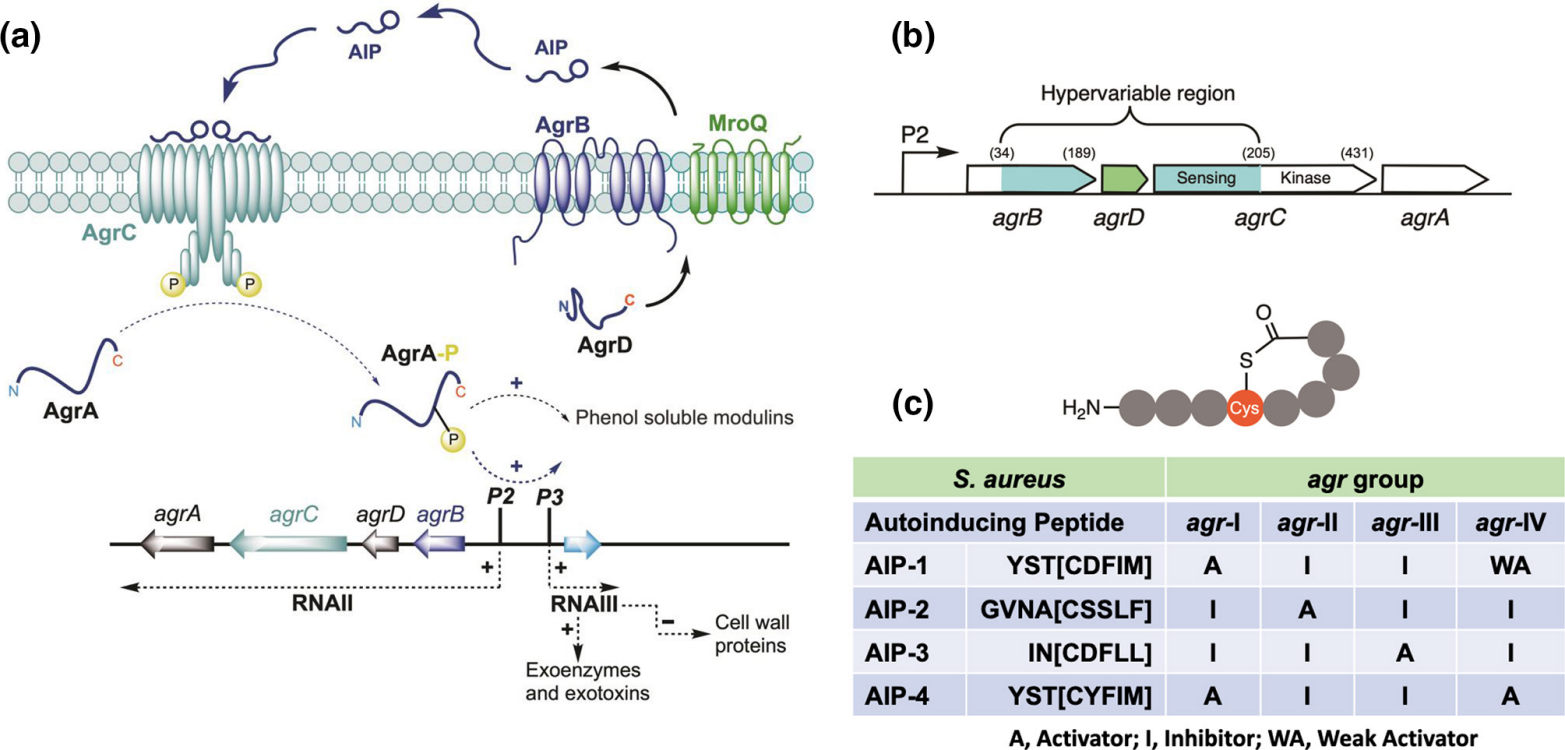
(**a**) Schematic of the staphylococcal *agr* quorum-sensing system. The *agrBDCA* locus is composed of two divergent transcripts, RNAII and RNAIII, driven by the *agr*P2 and *agr*P3 promoters, respectively. AgrD, the pro-peptide precursor of the autoinducing peptide (AIP) is processed at the cytoplasmic membrane by AgrB and MroQ such that AIPs are released extracellularly. AIPs bind to and activate the AgrC receptor, a membrane-bound histidine sensor kinase resulting in phosphorylation of the response regulator AgrA and activation of the *agrP2* and *agrP3* promoters. This drives the autoinduction circuitry to generate more AIP signal molecules and induces expression of virulence genes either indirectly via RNAIII or directly via the target gene promoters. (**b**) Schematic showing the hypervariable region (in green/cyan) of the *agrBDCA* locus incorporating *agrD* and giving rise to the different *agr* groups. Amino acid residues marking the beginning and ends of the variable regions are numbered (adapted from [142]). (**c**) Generalized AIP structure and summary table showing the amino acid sequences of the AIPs belonging to each of the four *S. aureus agr* groups and their cross-group activities. The brackets denote the amino acid residues within the macrocycle. In *S. aureus *, the AIP N-terminal tails have two, three or four amino acid residues.

In *S. aureus* the *agr* locus consists of two divergent transcriptional units, *agrBDCA* and the regulatory RNA effector, RNAIII controlled by the P2 and P3 promoters, respectively [20, 23]. (Fig. 2a). The P2 operon consists of four genes, *agrBDCA*, which are required for the activation of transcription from the P2 and P3 promoters while the P3 transcript, RNAIII, a 517-nucleotide transcript, is itself the primary effector for the *agr* response and also encodes δ-haemolysin [24]. AgrA and AgrC constitute a two-component system in which the transmembrane protein AgrC is the histidine sensor kinase and the cytoplasmic AgrA protein is the response regulator [23, 25]. The diffusible *agr* QS signal is an autoinducing peptide (AIP) (Fig. 2) derived via the AgrB-dependent proteolytic processing of the ribosomally synthesized AgrD pro-peptide. AIPs induce the *trans*-auto-phosphorylation of AgrC, which transfers the phosphate to a conserved Asp on AgrA. This binds to the *agrP2* promoter upregulating *agrBCDA*, conferring a positive-feedback loop that autoinduces AIP production and drives target gene expression directly via AgrA or via the AgrA-dependent *agr*P3 promoter and the post-transcriptional regulator, RNAIII [24] (Fig. 2a). There are four allelic variants (I–IV) of the *S. aureus agr* locus with respect to a hypervariable region contained in the *agrB*, *agrD* and *agrC* genes (Fig. 2b, c). These reflect the amino acid sequence variations in the four *S. aureus* AIPs and explains their specificity for AgrC as activators of their cognate receptors but competitive inhibitors of the other AgrC variants [20, 26–31]. For example, AIP-1 is a competitive antagonist of the AIP-2/AgrC2 and AIP-3/AgrC3 interactions whereas AIP-2 and AIP-3 antagonize AIP-1/AgrC1 interactions (Fig. 2c). AIP-4, which differs from AIP-1 by a single amino acid residue (Asp is replaced by Tyr; Fig. 2c), is an agonist of both AgrC4 and AgrC1 but an inhibitor of AgrC2 and AgrC3 [28, 29]. Recently Raghuram *et al*. [32] developed a software tool (AgrVATE; github.com/VishnuRaghuram94/AgrVATE) to interrogate *S. aureus agr* variability and evolution. Analysis of ~40 000 *S *. *aureus* genomes revealed that the distribution of the four *agr* groups is ~60 % group I, ~22 % group II, ~14 % group III and ~2.5 % group IV [32]. This extensive *in silico* analysis did not uncover any novel *agr* groups, intermediate AIPs or AIPs acquired from CONS. Potential relationships between certain infectious diseases and *agr* group have been highlighted such as between toxic shock and scalded skin syndromes and *agr* groups III and IV, respectively [33]. However, the number of strains examined was relatively small and others [34] found no associations between *agr*-specific groups and infection type. A recent major study found that the distribution of *agr* groups in over 10 000 strains from blood, skin and the nasal cavity was similar to the general distribution of *agr* groups [32]. *agr* acts as an AIP-dependent autoinducible system such that mutation of any of the corresponding *agrBDCA* genes results in the loss of activity [20]. As a QS-dependent master virulence gene regulator, it is also subject to control via by a sophisticated interconnected network of regulators, which integrates diverse environmental and host cues via two component sensor regulator systems such as SaeRS, SrrAB and ArlRS, sigma factors (e.g. SigB) and the SarA family (e.g. SarA, SarR, SarS, MgrA and Rot). A detailed consideration of the molecular nature and function of these gene regulatory elements in controlling *agr* is beyond the scope of this review and the reader is referred to recent reviews [23, 24].

### AIP identification and quantification

In the staphylococcal *agr* system, AIP signal molecules are thiolactones, which have similar structures but different primary amino acid sequences. Each AIP has a common central Cys residue, the thiol of which is linked to the α-carboxyl group of the C-terminal residue forming a five residue, 16-membered macrocycle with an exocyclic N-terminus of variable length (Fig. 2c). These have been identified by liquid-chromatography (LC) mass spectrometry (MS) aided by the annotated AgrD amino acid sequence data [27, 28, 35, 36] or by chemo-selective trapping [37]. AIPs have been produced by solid-phase chemical synthesis [28, 38] and via protein engineering using mini-intein technology [39]. They can readily be detected at nanomolar concentrations in cell-free *S. aureus* culture supernatants using cell-based transcriptional reporter assays for AIP-dependent AgrC activation or inhibition. Several such reporter assays have been described in which the *agrP2* or *agrP3* promoter is fused to a reporter gene such as *blaZ*, *gfp, lux* or *gluc* to provide colorimetric, fluorescence or bioluminescence outputs [26, 31, 40–42]. The assays can be conducted using microtitre plates to quantify AIP levels, evaluate AIP structure activity relationships (SAR), pharmacological properties (agonist, inverse agonist, antagonist) or the functionality of mutations in AgrC receptor proteins. For example, Jensen *et al*. [31], deleted the chromosomal *agr* locus and replaced it with the *luxCDABE* operon under the control of the *agrP3* promoter. The *agrA* and *agrC* genes were then introduced on a plasmid under the control of the *agrP2* promoter. Since this reporter system is unable to produce AIPs and lacks the autoinduction pathway characteristic of the native *agr* QS system, it facilitates the in-depth pharmacological evaluation of AIP analogues without interference from an endogenously active *agr* system [31]. It also facilitates the introduction of the desired native or mutated *agrC* gene and requires no additional reagents. For example, using this reporter for AIP-1/AgrC1, an EC_50_ of 6±1 nM was derived from a dose–response curve and an EC_50_ of 9±1 nM for AIP-4/AgrC4. In contrast, AIP-1 is a very weak activator of AgrC4 (EC_50_ 3542±997 nM) [31]. Comparable nanomolar EC_50_s have been reported by others for activation of AgrC by the cognate AIP [28, 29, 38] using alternative transcriptional reporters.

### AgrD pro-peptide processing by the transmembrane protein, AgrB

**Fig. 3.**
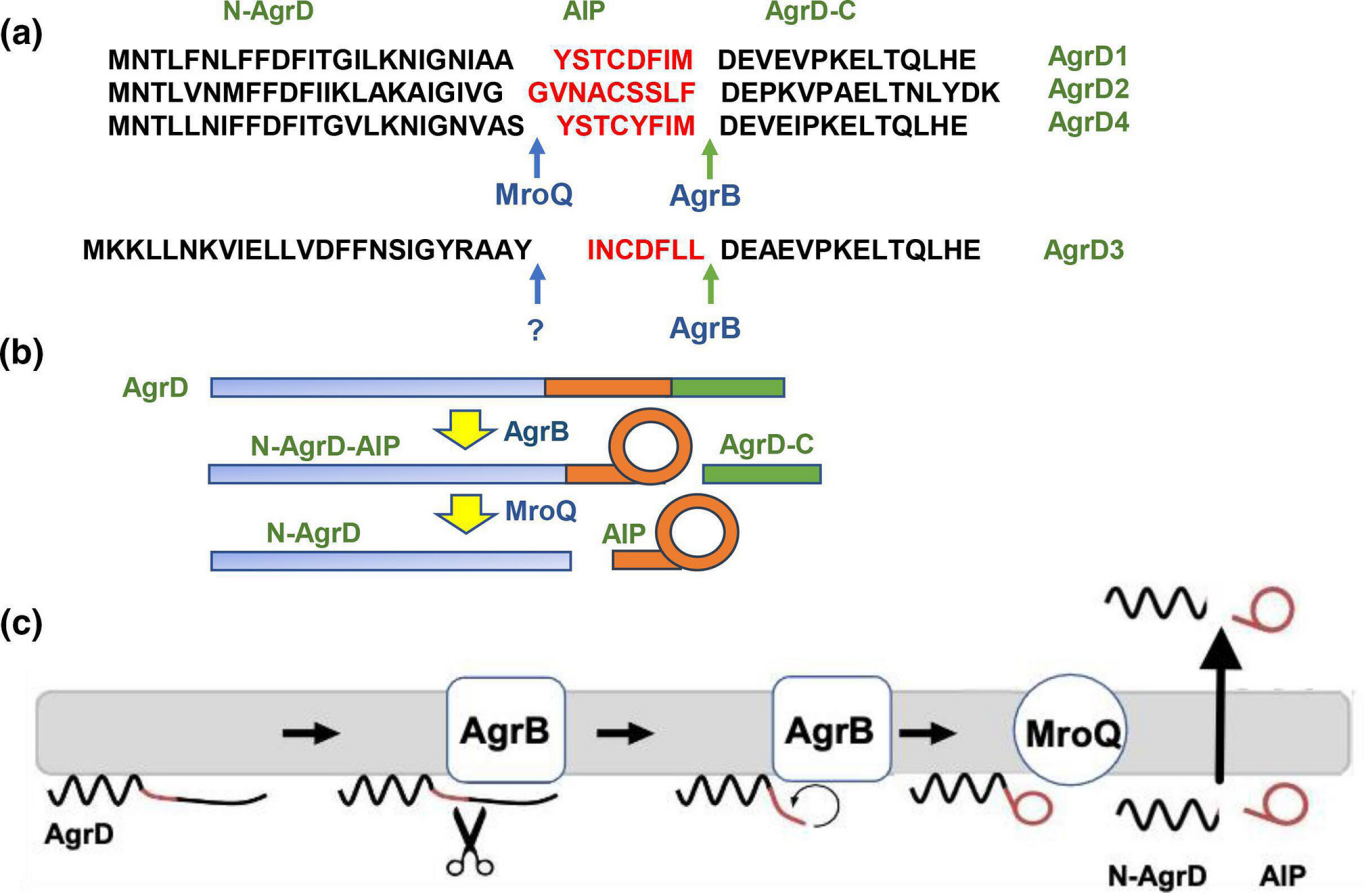
Schematic showing the processing of AgrD pro-peptides to generate the active cyclic AIP signal molecules. (**a**) Amino acid sequences of *S. aureus* AgrD1-D4 showing the AgrB cleavage site and the MroQ sites for AgrD1, D2 and D4. MroQ does not cleave AgrD3. (**b**) Processing of AgrD by AgrB and MroQ to release N-AgrD and the cyclic AIP. (**c**) Schematic showing the formation and release of the AIP and N-AgrD at the cytoplasmic membrane. Cleavage of AgrD by AgrB releases a 14 amino acid C-terminal peptide (AgrD-C), which is degraded in the cytoplasm. N-AgrD-AIP is cleaved by MroQ to release N-AgrD and the mature AIP. The mechanism by which the AIP and N-AgrD are exported is not known.

AIPs are derived from an internal fragment of an AgrD pro-peptide, which consists of 40–50 amino acid residues incorporating an N-terminal amphipathic leader (N-AgrD; 24–25 amino acids), a mid-region of 7–9 amino acid residues that constitutes the AIP and a charged C-terminal tail (AgrD-C; 14–15 amino acids) (Fig. 3). The generation of an extracellular AIP requires at least four membrane-associated steps (i) removal of AgrD-C, (ii) formation of the thiolactone macrocycle, N-AgrD-AIP (iii) cleavage of N-AgrD and (iv) export of AIP and N-AgrD (Fig. 3).

*S. aureus agrB* deletion mutants are unable to activate *agr* as they fail to produce AIPs. This is because AgrB, a unique transmembrane cysteine protease is required for processing the AgrD pro-peptide [27] via steps (i) and (ii) (Fig. 3). Although AgrB lacks homology with other proteins, the alignment of AgrB sequences from diverse Gram-positive bacteria has revealed multiple, highly conserved amino acid residues. However, the *S. aureus* AgrBs do exhibit some substrate specificity. AgrB1 is able to process AgrD1 and AgrD2 but not AgrD3 and vice versa. Chimeric AgrB1 and AgrB2 proteins have been constructed and regions putatively involved in AgrD group specificity identified [43].

Two of the invariant residues in AgrB homologues are Cys84 and His77. These appear to constitute a catalytic dyad that enables AgrB to cleave AgrD-C from AgrD. This is likely to result in the formation of an acyl-enzyme thioester intermediate, followed by peptidyl transfer to generate the thiolactone macrocycle via the internal Cys28 of AgrD so releasing N-AgrD-AIP (Fig. 3). Furthermore, an N-terminal amphipathic helix, the length of the C-terminal tail and certain C-terminal residues (e.g. E34 and L41; Fig. 3) of AgrD all appear to be essential for substrate recognition and processing by AgrB in bacterial cell-based assays [44–46].

When purified, the *S. aureus* AgrB protein forms stable dimers and is enzymatically active only when embedded in lipid bilayers [47]. It clearly drives a proteolytic cyclization reaction removing the C-terminal 14–15 amino acid residues of AgrD while reversibly catalysing the formation of a thiolactone ring, which undergoes slow irreversible hydrolysis to the corresponding linear form. The N-AgrD-AIP thiolactone is protected from ring opening by association with membrane phospholipids and the reaction is driven efficiently by the rapid degradation of the AgrD-C fragment [47], possibly via ClpP or ClpX in staphylococcal cells [48]

**Fig. 4.**
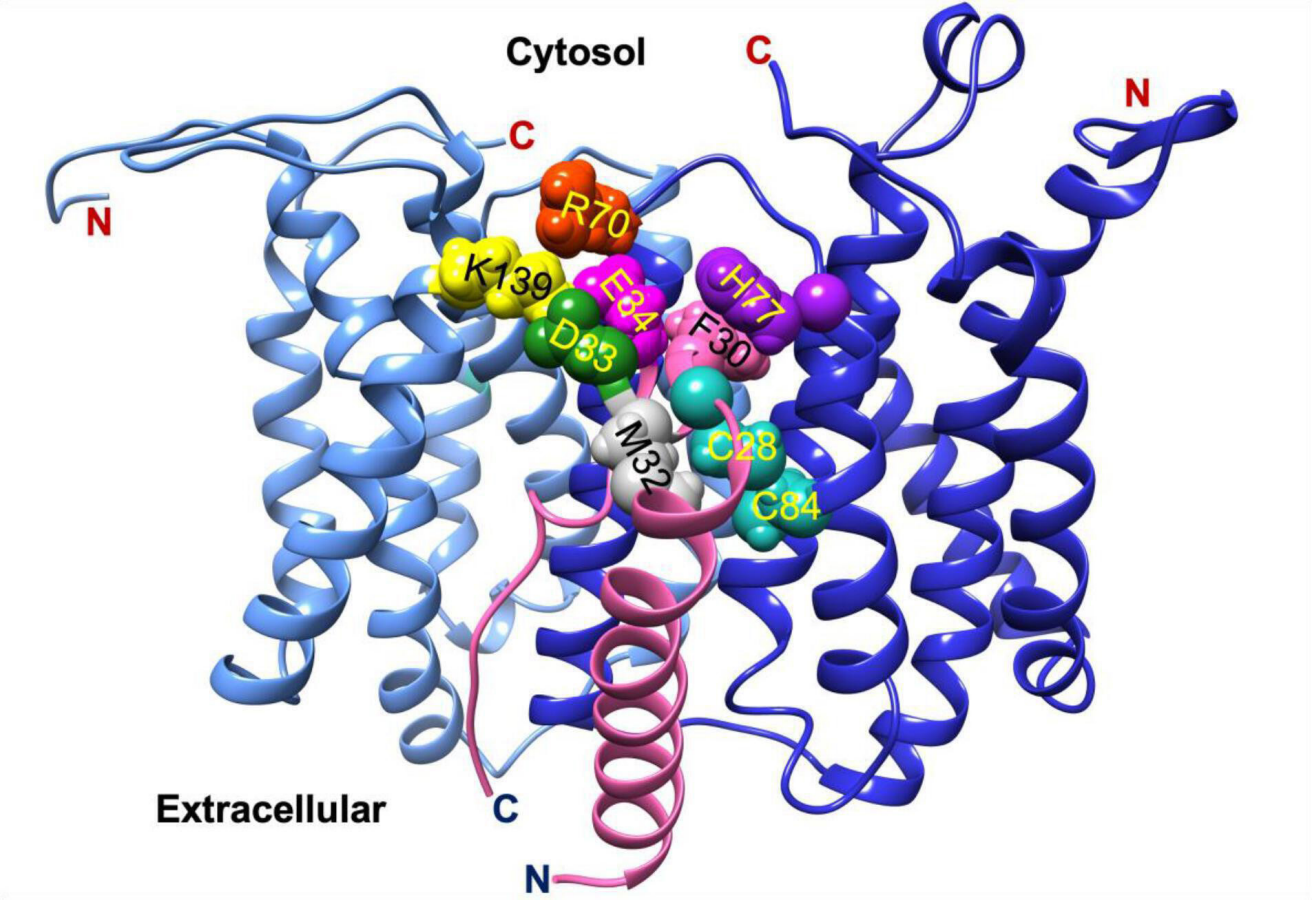
Conformation of membrane-embedded ternary complex AgrB_2_/AgrD after molecular dynamics simulations. The two AgrB proteins are inequivalent with AgrB-I (cornflower blue) guiding substrate AgrD (fuchsia) to the active site involving catalytic AgrB-II (blue). AgrD residues C28 and M32 are close to each other and to catalytic AgrB-II C84. Key interactions stabilizing the complex include AgrB-I K139-D33 and AgrB-I R70-E34 contacts with AgrD, and AgrB-II H77-F30 *via* π-interactions. Sidechains are colour-coded aqua (C28, C84 AgrB-II), grey (M32 AgrD), pink (F30 AgrD), purple (H77 AgrB-II), fuchsia (E34 AgrD), green (D33 AgrD), yellow (K139 AgrB-I) and orange (R70 AgrB-I). All four AgrB termini are on the cytosolic side (top) consistent with six transmembrane domain topology of AgrB. The (AgrB)_2_/AgrD complex orientation here is shown with cytosol at the top offering a better view of the cytosol-accessible active site (adapted from [51]).

Topology predictions for AgrB initially proposed two different models; a six transmembrane domain model with both termini at *cis* [44, 49] and a four transmembrane domain with an additional half-transmembrane hairpin where both termini are at *trans* [50]. These contradictory models, which contain structurally implausible domains, have yet to be replaced by high-resolution crystallographic or NMR-derived structures. However, a recent molecular dynamics model supported by experimental circular dichroism data revealed a tightly packed six transmembrane domain helical topology for AgrB with both N- and C-termini on the same side and placing amino acid residues known to be important to function on the *cis* side near the polar/apolar interface [51]. These include the enzyme active site Cys84, which is located in the membrane interior providing access to the AgrD substrate. Additional molecular dynamics simulations of AgrB and AgrD in a membrane environment have provided a new model incorporating an AgrB dimer with crucially two non-equivalent AgrB sites in which one AgrB monomer facilitates insertion and positioning of AgrD in the correct orientation for catalytic processing by the second AgrB (Fig. 4) [51]. Whether the proposed (AgrB)_2_AgrD complex exists requires further experimental confirmation.

### N-terminal cleavage of AgrD requires a second transmembrane protease, MroQ

The identity of the transmembrane protease required for cleaving the N-terminal region from the *N*-AgrD-AIP thiolactone to release the AIP [step (iii) (Fig. 3)] has been something of an enigma. Expression of *agrB* and *agrD* from *agr* group I in *Escherichia coli* surprisingly resulted in the formation of extracellular AIP [45] indicating that *E. coli* must also possess an AgrD N-terminal cleaving protease. Kavanaugh *et al*. [52] developed a fluorescence assay based on a linear synthetic peptide designed to identify *S. aureus* peptidases that could potentially cleave the AgrD N-terminal amphipathic peptide. Evidence was provided in support of signal peptidase B (SpsB) as the missing transmembrane protease, which included the ability of SpsB peptide inhibitors to reduce *agr* reporter expression and AIP production in *S. aureus *. These data were also consistent with AIP production in *E. coli *, which has only one, essential signal peptidase that, in a temperature-sensitive mutant, can be genetically complemented by SpsB. However, more recent work indicates that in *S. aureus* a different transmembrane protein is the primary enzyme involved in removing AgrD N-terminal peptides [53, 54].

**Fig. 5.**
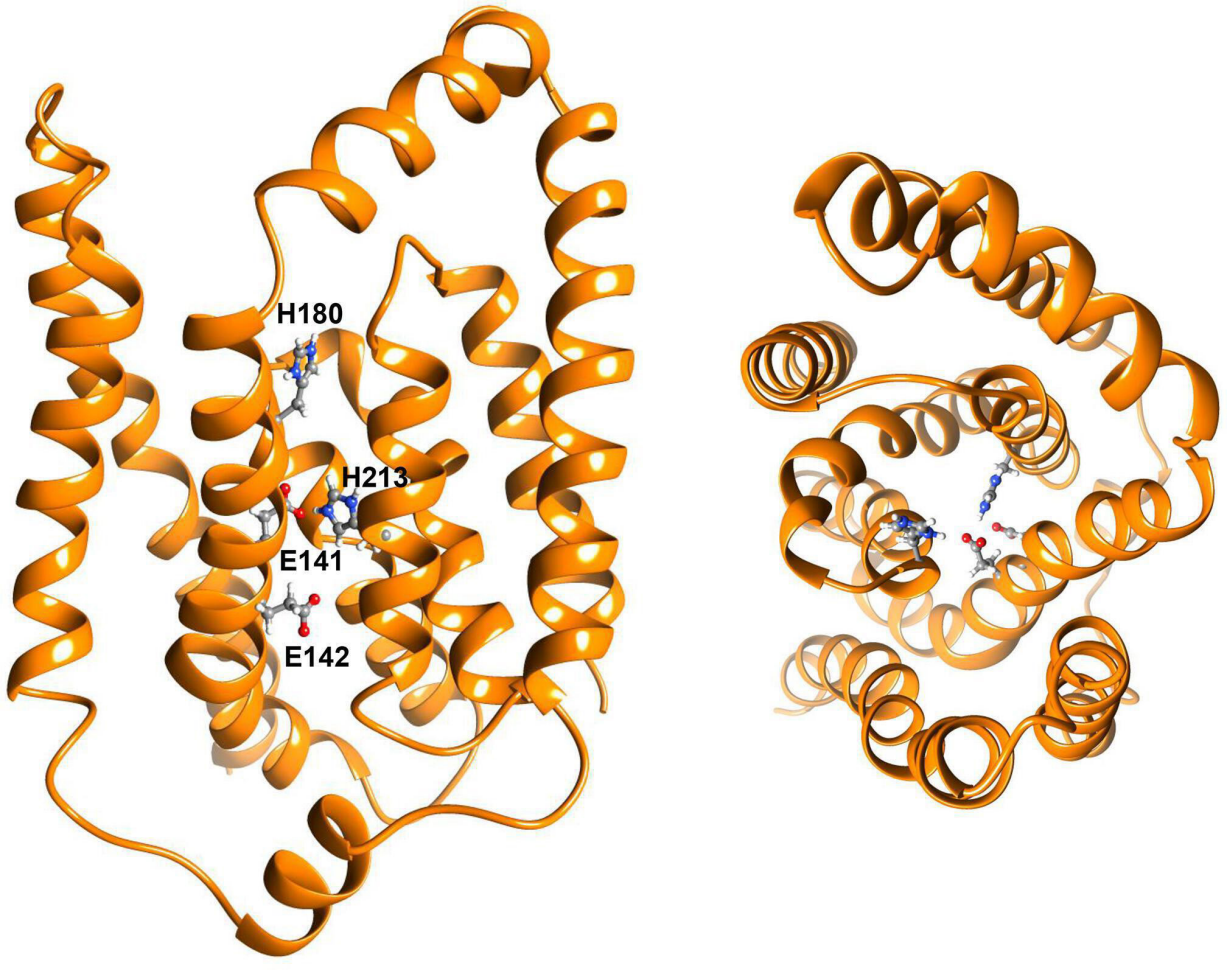
Topological model of transmembrane endopeptidase MroQ. The homology model of MroQ (unpublished) was obtained from I-TASSER [143] and annealed in atomistic MD simulations using NAMD [144] within a membrane patch built in CHARMM-GUI [145]. The model reveals an eight TMD topology – lateral view (left); and axial view (right). Residues Glu141 and Glu142 and His180 and His 213 required for MroQ functionality are shown within the membrane region where they have access to the hydrophobic AgrD substrate.

Upstream of the *agr* locus, a gene termed *mroQ*, the mutation of which results in the loss of virulence and significantly reduced extracellular AIP levels was identified [53, 54]. The translated product is predicted to have eight transmembrane-spanning helices (Fig. 5) and is a member of the CPBP (CAAX proteases and bacteriocin-processing enzymes) family of putative membrane metalloproteases encompassing over 5000 members that incorporate the misnamed ‘Abortive phage infection’ (Abi) domain [55]. CPBP superfamily members share three signature motifs and are found in both prokaryotes and eukaryotes. In the latter, prenylation is crucial for the function and membrane protein targeting [55]. In bacteria, only a few CPBP proteins have been studied experimentally but their functions in general have remained relatively elusive. SkkI for example from *Lactobacillus plantarum* functions as a bacteriocin receptor and immunity protein [56], whereas the group B streptococcal CPBP protein AbxI interacts with the histidine sensor kinase, CovS to control virulence gene expression [57]. For SkkI but not for AbxI proteolytic activity is necessary for function. Several CPBP proteins are present in *S. aureus* including MroQ, which contains the EEXXXR and FXXXH motifs considered necessary for enzyme activity. The critical residues for metalloprotease activity include the predicted MroQ catalytic site Glu141 and Glu142 and the His residues 180 and His213 required for zinc co-ordination [53] (Fig. 5). Replacement of either Glu or the His with Ala in these motifs variably reduced AIP production but *S. aureus* virulence was only attenuated in a mouse skin and soft-tissue infection model to the same level as an *agr* deletion mutant for Glu141Ala replacement [53]. In a cell-based assay, expression of *agrD* in an *S. aureus mroQ* mutant resulted in the accumulation of a membrane-associated AgrD intermediate but no AIPs were produced [58]. The amino acid sequence of MroQ does not vary with *agr* group suggesting that the same enzyme processes all four *agr* groups. However, in *agr* group III strains, deletion of *mroQ* did not prevent the production of an active AIP [58]. Whether AgrB and MroQ interact directly in *S. aureus* membranes to process AgrD in a manner analogous to that of the MroQ homologue SpdC (LyrA) with SagB is not known. SpdC and SagB (a membrane-bound *N*-acetylglucosaminidase) form a complex that functions as a peptidoglycan release factor. However, the conserved residues required for CAAX proteolytic activity are not required by SpdC [59, 60].

Biochemical confirmation that MroQ is able to remove the N-terminal peptide from N-AgrD-AIP was obtained by reconstituting the MBP-MroQ fusion proteins into proteoliposomes and incubating with N-AgrD-AIP thiolactones from *agr* groups I, II and III. The efficient generation of AIP-1 and AIP-2 was confirmed by LC MS/MS, although no cleavage of the *agr* group III substrate was observed. By including AgrB in the proteoliposomes with MroQ, complete reconstitution of AIP biosynthesis from AgrD was achieved [61]. These elegant biochemical studies together with the observation that SpsB is unable to cleave AIP biosynthetic intermediates *in vitro* confirmed that MroQ is the primary transmembrane protease involved in N-terminal cleavage of the AgrB processed AgrD thiolactone product. However, although the *agr* N-AgrD-AIP thiolactones from *agr* groups I and II were substrates for MroQ, it did not cleave the *agr* group III AgrD peptide thiolactone. Thus, there must be an alternative enzyme, which does not appear to be SpsB.

Both the N-AgrD peptide and AIP are released from the cells into the extracellular environment. N-AgrD has PSM toxin-like properties and is also amyloidogenic, capable of forming amyloid fibrils in *S. aureus* biofilms [62]. Gonzalez *et al*. [63] identified both formylated and non-formylated peptide variants derived from N-AgrD in *S. aureus* culture supernatants, which were cytotoxic for mammalian cell lines, modulated neutrophil chemotaxis and increased the size of murine skin lesions induced by an *S. aureus agr* mutant. Whether MroQ processes N-AgrD-AIP thiolactones on the cytoplasmic or external face of the cytoplasmic membrane has not yet been established. In either case it is not clear whether export occurs via MroQ, AgrB, an MroQ/AgrB complex or via dedicated transport mechanisms such as the ABC transporters PmtCD and AbcA [64]. These can independently export PSMs from either membrane or cytosolic environments.

### The AgrCA two-component sensor regulator system

**Fig. 6.**
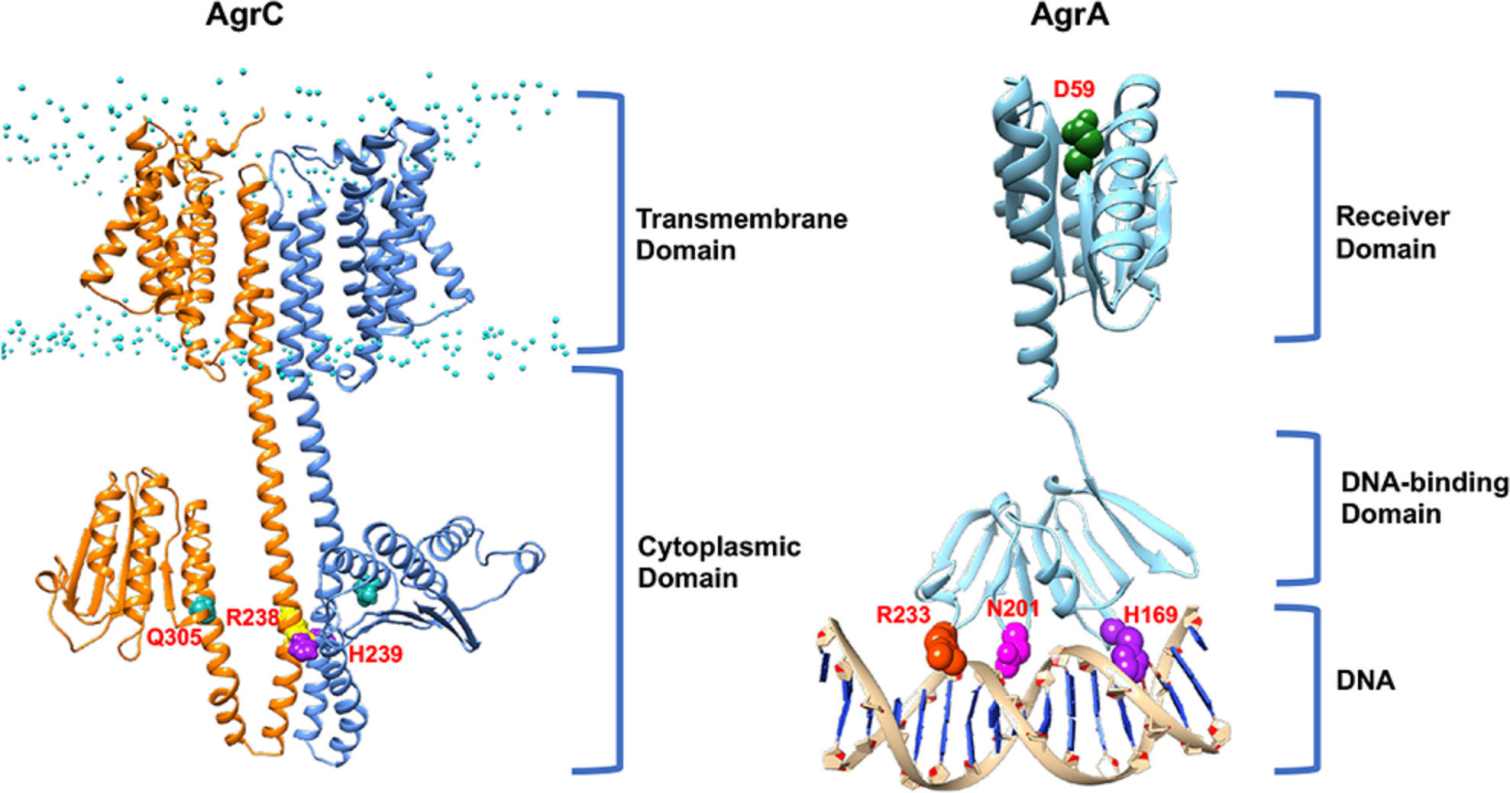
Architecture of the AgrC and AgrA two-component sensor and response regulator proteins. Models of the homodimeric sensor kinase AgrC (left) (orange/cornflower blue) and response regulator AgrA (right) (cyan) associated with the *agr*P2 promoter site. Initial conformations of AgrC and AgrA were obtained from AlphaFold [146]. The AgrC dimer was built using ClusPro [147]. Lipid phosphates are shown to mark the membrane interface (P atoms are shown in cyan). His239, responsible for AgrC autophosphorylation, is shown in purple; Arg238 (yellow) and Gln305 (fuchsia) are important for the molecular ‘latch’ that stabilizes AgrC in the ‘off state’ [67]. The AgrA model was superimposed onto the structure of the AgrA C-terminal DNA-binding domain [PDB:3BS1] [71]. Asp59 (green) phosphorylation by AgrC is important for activation of AgrA. His169 (purple), Asn201 (fuchsia) and Arg233 (orange-red) are responsible for DNA recognition.

Once exported, AIPs are sensed via a two component system (TCS) in which AgrC is the histidine kinase sensor and AgrA, response regulator (Fig. 2a). AgrC belongs to the minor HPK10 histidine kinase protein sub-family [65] and has a modular architecture with an N-terminal sensory module incorporating an AIP-binding site linked via a helical linker to a C-terminal cytoplasmic module containing two subdomains [25] (Fig. 6). These are the dimerization and histidine phosphorylation (DHp) and the catalytic and ATP-binding (CA) subdomains [25]. The CA subdomain containing the G1 box lacks both the conserved DXGXG motif and the key Asp residue, which is replaced by Asn such that the binding affinity of AgrC for ATP and hence kinase activity is likely dependent on cellular energy levels [25, 66]. The interactions between AgrC and the activating AIP ligand are highly specific occurring at nanomolar affinities where the AIP non-cooperatively binds to AgrC in a 2 : 2 stoichiometry [25].

AgrC behaves like a rheostat, where activation of its membrane spanning domain following the binding of an AIP results in the twisting of a helical linker relative to the cytoplasmic domain and subsequent dimerization that results in AgrA phosphorylation [25]. The binding of a classical competitive antagonist such as AIP-3 to AgrC1 blocks rotation of the helical linker whereas AIP-2, an antagonist that behaves as an inverse agonist of AgrC-1 drives rotation of the helical linker in the opposite direction to that of the agonist AIP-1 [25].

A key non-covalent interaction between Arg238 and Gln305 that stabilizes AgrC in the ‘off’ state has also been identified [67] (Fig. 6). This ‘latch’ is proposed to lift following the binding of the cognate AIP to the extracellular AgrC sensor domain, thus mediating the structural changes that result in AgrC activation. Replacement of Arg238 with Ala renders AgrC constitutively active. The mechanism by which AIPs induce the structural changes in AgrC that lead to activation of kinase activity is not yet clear, although AIP-binding is likely to either induce or stabilize specific rotational conformations [67].

No full-length AgrC structure has yet been obtained and hence the nature of the AIP-binding site is currently not known. However, topology models predict that the N-terminal sensory module of AgrC contains six transmembrane-spanning helices and three extracellular loops (Fig. 6). Since the *S. aureus agr* system has undergone significant evolutionary divergence, the retention of functionality requires that changes in AgrD, which modify the AIP structures should be accompanied by compensatory changes in the AgrC receptor protein. Since AIPs are not internalized, the AIP-binding site is likely to be on the outer face of the transmembrane AgrC protein and therefore likely to involve the extracellular loops. AIP-1 and AIP-4 differ by a single amino acid; when the corresponding AgrC1 and AgrC4 are compared, only two of their three predicted AgrC extracellular loops exhibit amino acid differences [31, 68]. In loop 1, 7 out of 19 and in loop 2, 3 out of 9 amino acid residues differ. Replacement by site-specific mutagenesis of these in AgrC4 either singly or in combination with those from AgrC1 revealed that while differential recognition of AIP-1 and AIP-4 depends primarily on three amino acid residues in loop 2, loop 1 was essential for receptor activation by the cognate AIP [31, 68]. Furthermore, a single mutation in the AgrC1 loop 2 resulted in conversion of (Ala_5_)AIP-1, a non-native AgrC1 inhibitor, to an activator, essentially resulting in the forced evolution of a ‘new’ AIP group [31]. Taken together, these data suggest that extracellular loop two may constitute the AIP macrocycle-binding site while the exocyclic N-terminal amino acids interact with loop 1 to facilitate receptor activation. However, there is no direct evidence to demonstrate that the key amino acid residues in AgrC loop 2 are directly involved in AIP-binding. It is conceivable that they could act indirectly on the conformation/presentation of direct contact residues elsewhere on AgrC [68]. The specificity of AgrC1 could also be further broadened by replacement of Ile at position 171 with Lys in the third predicted AgrC extracellular loop. This site-specific AgrC1 mutant was activated at nM EC_50_ concentrations not only by the cognate AIP-1 but also by AIP-3, AIP-4 and (Ala_5_)AIP-1 [69]. Multiple AgrC mutants, which are constitutively active have also been isolated and map to both the last transmembrane helix of the sensor domain and to the histidine kinase domain [69].

Once AgrC has been *trans*-autophosphorylated, phospho-transfer to AgrA, a member of the LytTR class of transcriptional regulators, occurs via the AgrC DHp cytoplasmic phospho-transfer sub-domain. AgrA consists of an N-terminal receiver domain containing the conserved Asp residue that drives dimerization upon phosphorylation and a C-terminal DNA-binding domain [70] (Fig. 6). Dimerization of AgrA enhances DNA binding to the LytTR domain-binding sites of which there are two, 9 base pair, high affinity sites in the *agrP2* promoter region separated by 12 bp and both a high- and a low-affinity LytTR binding site in the *agrP3* promoter region [71]. This enables differential expression of RNAII and RNAIII to facilitate QS via autoinduction and to avoid premature expression or degradation of RNAIII [46, 72]. Related *agr* promoter region motifs have also been identified in genes directly regulated by AgrA such as those coding for PSMs [73].

### AIP-mediated activation and inhibition of AgrC

**Fig. 7.**
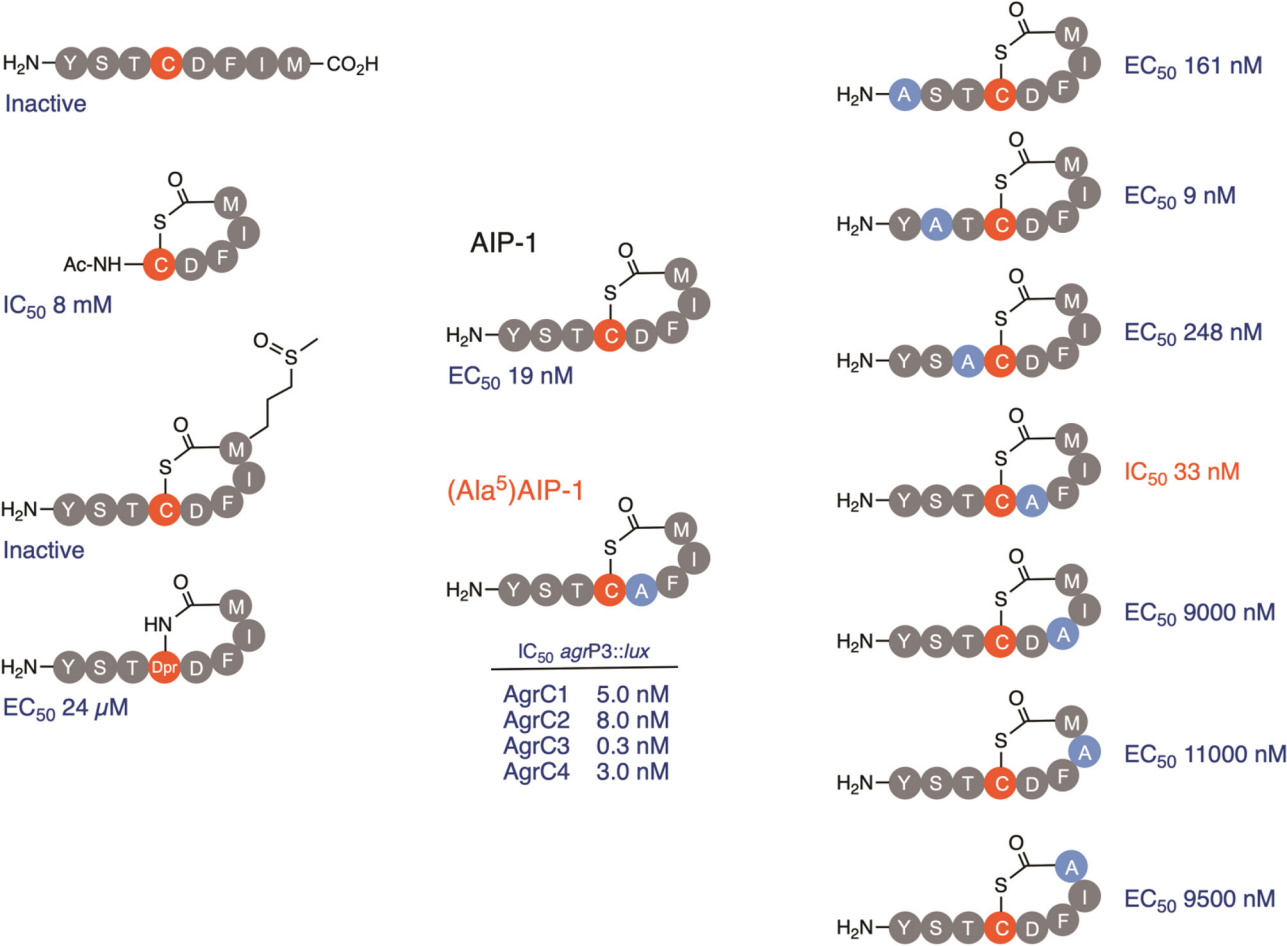
SAR for *S. aureus* AIP-1 showing how minor modifications to the peptide sequence including Ala-scanning influence activity. Substitution of the Asp residue (**d^5^**) with Ala to give (Ala^5^)AIP-1 converted AIP-1 from an activator of AgrC1 to a potent cross group inhibitor. IC_50_ data from [29].

Extensive AIP structure activity (SAR) studies using transcriptional reporters in *S. aureus* in combination with native AIPs and diverse AIP analogues have established the key molecular features for AgrC activation and inhibition [22, 28–30, 74–77] (Fig. 7). In general, minor differences in the AIP peptide sequence result in the complete loss of agonist activity. However competitive inhibition is highly tolerant of AIP sequence variation [22]. The macrocyclic ring is essential for AgrC activation as the corresponding linear peptides are inactive [26, 27, 78]. Similarly changes to the size of the macrocycle are not tolerated [79]. Removal of the three exocyclic N-terminal amino acids in AIP-1 also results in the loss of agonist activity as does replacement of the thiolactone S with N or with O to form the corresponding lactam (Fig. 7) and lactone, respectively [28, 29]. For AIP-1, replacement of each l-amino acid residue in turn with the corresponding d-isomer resulted in six out of the eight analogues exhibiting markedly lower activity. However, exchanging either the macrocycle Phe or the terminal Met residue with the corresponding d-isomer had little impact suggesting that the AIP-binding site in AgrC1 is able to accommodate these differences [28]. S-oxidation of the methionine thioether side-chain to form the methionyl sulphoxide derivative inactivated AIP-1 (Fig. 7), as did replacement of the Met with norLeu, Ser, Glu, Lys or Pro but not Ile, further emphasizing the critical role of the C-terminal thioether side-chain for AgrC1 activation [28, 29]. AIP-4, which also has a Met in the same position as AIP-1, is the only other *S. aureus* AIP capable of being inactivated via formation of the methionyl sulphoxide [28]. Substituting each AIP-1 amino acid residue in turn with Ala (Fig. 7), except for the central Cys, revealed that the only replacement showing increased activity (by ~2 fold) was for the exocyclic Ser [28]. The other AIP-1 Ala analogues were either inactive or exhibited reduced agonist activity, except that replacement of the endocyclic amino acid residue (Asp) located C-terminally to the central Cys with Ala converted AIP-1 from an activator to a potent low nanomolar IC_50_ cross-group inhibitor [28, 29] (Fig. 7) In AIP-4, the endocyclic Asp residue is replaced by Tyr (Fig. 2) and is therefore the critical determinant of AIP specificity for *agr* groups I and IV [28, 29]. Detailed SAR studies of activation and inhibition of the cognate AgrCs have also been undertaken for AIP-2 and AIP-3 with broadly similar findings to those reported for AIP-1 and AgrC1 [22, 29, 30, 38, 74–77]. These SAR data suggested that the macrocycle was required for receptor recognition and binding while the exocyclic region was necessary for receptor activation [22, 80]. Further refinement of these data via solution NMR structural analysis of the native AIP-3 peptide and a series of analogues revealed the importance of a hydrophobic ‘bulge’ formed by hydrophobic endocyclic residues and exocyclic tail contacts [22, 75]. These findings have highlighted the contribution of 3D conformation and the orientation of the AIP exocyclic tail relative to the macrocycle with respect to AgrC activation and inhibition.

### *S. aureus agr* heterogeneity and environmental inactivation

The *agr* system plays a key role in reciprocally regulating planktonic exotoxin producing, colonization and biofilm-associated lifestyles of *S. aureus *. It functions as a positive feedback loop displaying bimodal, heterogeneous behaviour that leads to the emergence of distinct subpopulations of *S. aureus* cells [81]. These subpopulations are characterized by the presence or absence of *agr* activity and by their relative numerical sizes consistent with bet-hedging strategies where some cells behave as individuals while others act co-operatively [82]. Such phenotypic heterogeneity has been also observed in the QS populations of other bacterial species where it may be transient and restricted to the early stages of activation with the population subsequently becoming homogeneous or heterogeneity may persist resulting in a bimodal, heterogeneous population [82].

In *S. aureus* the ratio of the *agr* ‘on’ to *agr* ‘off’ sub-population may be modified in response to environmental signals such as higher Mg^2+^ concentrations that increase cell wall rigidity by binding to teichoic acids and triggering the σ^B^-mediated down-regulation of *agr* [81]. Other cell-wall changes have been observed to modify *agr* activation. For example, in some, but not all, HA-MRSA strains [83, 84] expression of *mecA,* which codes for penicillin-binding protein 2A, reduced *agr* expression and virulence in a mouse infection model. Expression of *agr* could be restored by partially digesting the cell wall, suggesting that MecA-induced changes in cell wall architecture potentially reduce accessibility of the AIP to AgrC. Regulatory interdependence of *mecA* and *agrA* has also been noted for certain CA-MRSA strains in which methicillin resistance is *agr*-regulated [85]. HA-MRSA and CA-MRSA strains appear to have similar kinetics of *agr* activation but the latter achieve much higher magnitudes [85].

For activation of *agr*, AIP levels must reach a critical threshold concentration. This is not fixed but will vary according to the relative rates of AIP production, accumulation, diffusion and inactivation, which in turn will also depend on the prevailing growth environment. Little difference in the EC_50_s for AgrCs activated by their cognate AIPs (all low nM) are apparent [28, 29, 38]. Exogenous provision of the cognate AIP at the time of inoculation overcomes the ‘quorum’, prematurely activating *agr* although there is a window within the first few hours of growth after which *S. aureus* does not respond [86, 87]. Genotype versus *agr* locus-dependent differences in *agr* dynamics have been investigated in the context of *agr* group divergence by constructing congenic *S. aureus* strains (8325–4 and Newman) each with a different *agr* group allele and carrying an *agrP3-blaZ* fusion [86]. These revealed differences in the timing and magnitude of *agr* activation with *S. aureus agr* group III cells showing the most delayed induction and lowest level of *agr* expression, which in turn was reflected in cell-wall protein and exotoxin production. Whether such differences impact on virulence in infection models has not yet been established.

The ability to switch *agr* on or off during different stages of infection of host cells and in different tissues is clearly advantageous when moving from an acute toxigenic to a chronic persistent lifestyle, where increasing production of surface adhesins, reducing exotoxin secretion and either taking up intracellular residence or forming a biofilm enables *S. aureus* to avoid host immune defences. *In vivo* evidence supporting this on/off *agr* switch was obtained by Wright *et al*. [88] using whole body luminescence imaging of *S. aureus* transformed with an *agr*P3-*lux* expression vector. This revealed early rapid activation of *agr* followed by several days without any *agrP3* expression prior to renewed light output. While this switching of *agr* may have been due to transient *agrP3* expression, it could alternatively have been a consequence of either exhaustion of the LuxAB and LuxCDE enzyme substrates FMNH_2_ and a long-chain fatty aldehyde or reduced oxygen availability in deeper tissues resulting in a lack of luciferase activity [89].

Although AIPs are not known to be degraded or inactivated by endogenous staphylococcal enzymes, the C-terminal Met of *S. aureus* AIP-1 is S-oxidized during aerobic growth in laboratory media to form the corresponding methionyl sulphoxide [28] (Fig. 7). This methionyl sulphoxide-containing compound is unable to activate or inhibit AgrC [28]. Whether its formation and accumulation impact on the timing of *agr* induction by reducing AIP-1 levels (or AIP-4 which is the only other *S. aureus* AIP with a terminal Met) below the activation threshold is not known. However, this *S*-oxidation reaction has *in vivo* relevance as it occurs in response to phagocyte derived reactive oxygen and nitrogen species such as hypochlorous acid (HOCl) and peroxynitrite (ONOO^-^) and results in down-regulation of *agr* and a concomitant reduction of virulence in a mouse skin infection model [15]. Oxidative stress can also down-regulate *agr* via AgrA modification as a consequence of disulphide bond formation between the redox-reactive Cys119 and Cys288 leading to dissociation of the modified AgrA from DNA [90]. In addition, Cys119 can undergo ‘CoAlation’, i.e. the covalent modification by co-enzyme A, which, under nutrient deprivation or oxidative stress conditions also reduces the affinity of AgrA for the *agrP2* and *agrP3* promoters [91]. During *in vitro* competition and evolution experiments, oxidative stress was observed to drive the emergence of *agr* mutants, which possess a fitness advantage only under aerobic growth conditions due to the reactive oxygen species generating capacity of PSMs and RNAIII-regulated factors [92]. Consequently, *agr* imposes an oxygen-dependent fitness cost such that hypoxia favours maintenance of QS and increased exotoxin production. This oxygen-driven tuning of the *agr* system may therefore exert a major influence on tissue-dependent disease progression during infection.

Exposure to air pollution and in particular particulates such as black carbon (BC) are associated with exacerbations of chronic respiratory disease [9]. Growth of *S. aureus* in BC prior to inoculation increased the adhesion to, and invasion of, human epithelial cells *in vitro* and murine respiratory tract colonization and pulmonary invasion *in vivo* [9]. Global transcriptional analysis revealed that numerous *agr*-regulated exoprotease, exotoxin and immune evasion genes were upregulated while certain adhesin and metabolic genes were repressed suggesting that that BC acts directly on the pathogen rather than exclusively on the host [9]. The mechanism by which BC controls this subset of the *agr* regulon has yet to be elucidated.

Host factors known to impact on the *agr*-driven switch from a colonizing to an invasive phenotype include serum proteins such the low-density (LDL) and very low-density (VLDL) particles associated apo-lipoprotein B (ApoB), which interfere with *agr*-dependent QS by specifically and reversibly sequestering *S. aureus* AIPs [93]. This ApoB-mediated downregulation of *agr* in serum can be circumvented using constitutively active AgrC variants [94]. All four *S. aureus* AIPs as well as the inactive methionyl oxide derivative of AIP-1 bind to ApoB via an interaction that is likely to be dependent on the hydrophobic thiolactone macrocycle as the (less hydrophobic) linear peptide corresponding to AIP-1 does not bind. The *in vivo* relevance of these findings is supported by the greater susceptibility of mice deficient in ApoB to invasive infection with a wild-type *S. aureus* strain compared with an isogenic *agr* deletion mutant [93].

**Fig. 8.**
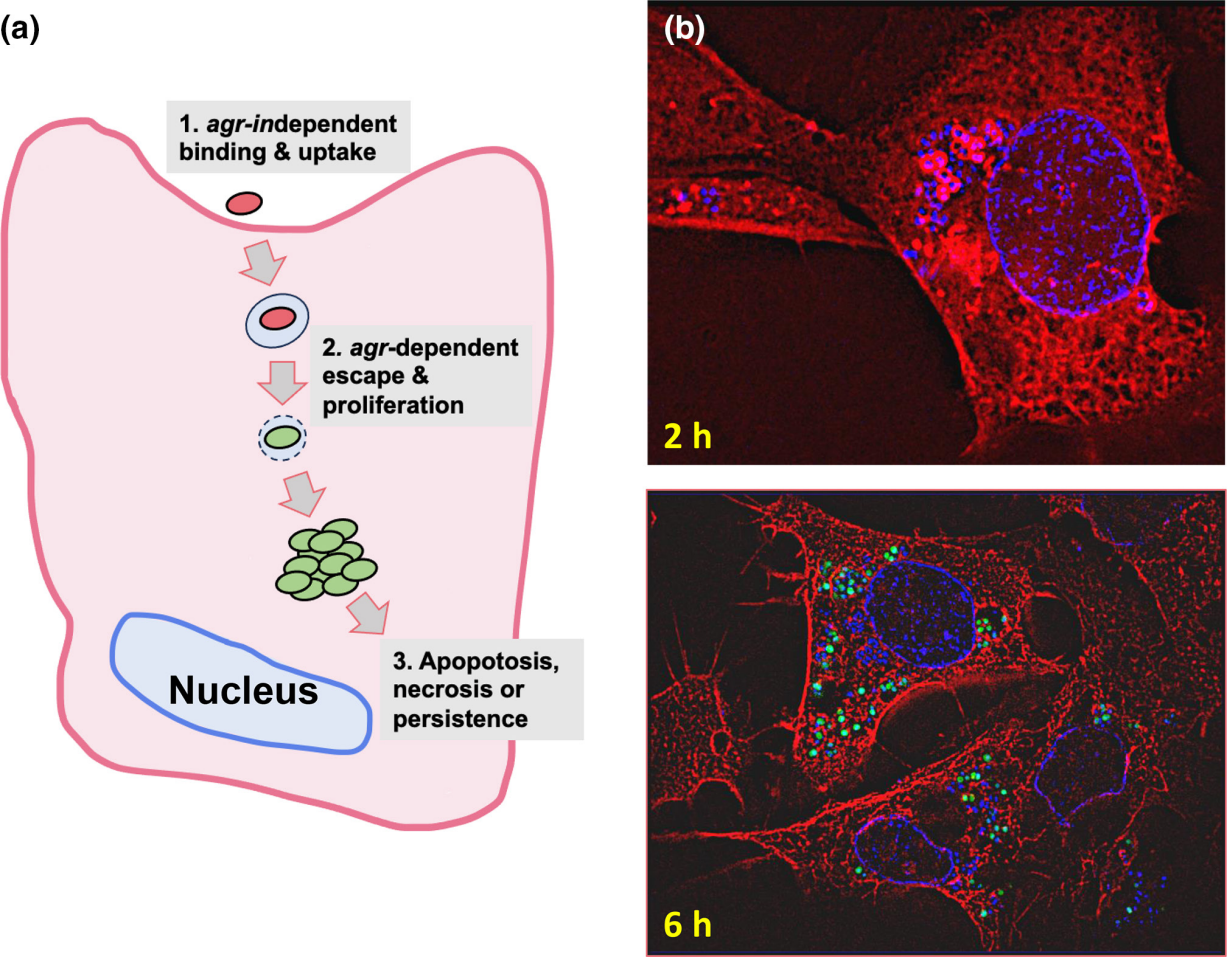
(**a**) Intracellular uptake of *S. aureus* into non-professional epithelial and endothelial cells is independent of *agr* expression. Once internalized, *agr* expression precedes endosomal escape by facilitating endosomal lysis via α-haemolysin or the PSMs. This enables *S. aureus* to replicate and persist within the cytoplasm protected from host immune defences and antibiotics or to lyse the host cells and establish further rounds of uptake and release leading to tissue destruction. (**b**) Fluorescence microscopy of *S. aureus* transformed with an *agrP3-gfp* reporter invading mammary epithelial cells and stained with DAPI (DNA; blue) and an anti-tubulin-Cy3 conjugate (red; microtubules). After 2 h incubation, intracellular staphylococcal cells are observed (white arrow) with red cell walls as the Cy3 antibody conjugate has bound to protein A showing that *agr* has not yet been activated. As the infection proceeds to 6 h *agr* expression has clearly been induced as observed by the high level of *gfp* expression (green) in the bacterial cells (adapted from [14]).

Although once considered to be an exclusively extracellular pathogen, *S. aureus* can internalize and survive in a variety of mammalian cells including endothelial, epithelial and professional phagocytic cells where they contribute to chronic and relapsing infections [95]. Quenching of *agr*-dependent QS in the bloodstream, for example, ensures that the *S. aureus* cell-wall proteins required for host cell attachment and internalization remain highly expressed [93]. Once inside an endosome or phagosome, *S. aureus* cells escape into the cytoplasm, kill the host cell, be killed or remain intracellular, protected from host defences and acting as a reservoir for persistent infections (Fig. 8). Within these intracellular vesicles, AIPs accumulate rapidly activating *agr* and leading to the expression of exotoxins such as α-haemolysin and the PSMs, which lyse the endosomal or phagosomal membrane [14, 16]. With respect to neutrophil phagosomes, AIP-mediated staphylococcal escape can be blocked by inactivation of AIP-1 and AIP-4 by S-oxidation via NAPDPH-derived oxygen radicals [15]. This intracellular escape process has been termed confinement or compartment sensing rather than QS since *agr* activation occurs in a single trapped bacterial cell rather than in a population [10, 96].

### *agr* dysfunction and infection

Despite the relevance of *agr* to *S. aureus* virulence in animal infection models, *agr* defective mutants are commonly found in clinical samples from both asymptomatic nasal carriage and serious infections where the loss of *agr* functionality in both MRSA and methicillin-sensitive (MRSA) strains is associated with greater adaptability, persistent infections and poorer outcomes [87, 97–103]. In healthy individuals in the community, nasal carriage of *agr* mutants has been reported to be relatively low at ~9 % colonization. However, *agr* dysfunction has been strongly associated with hospitalization and antibiotic usage suggesting a trade-off between virulence and antibiotic resistance [100, 102]. *S. aureus agr* mutants exhibit enhanced survival in the presence of daptomycin because they shed phospholipids that neutralize the antibiotic extracellularly. In contrast, *agr*-functional strains released less phospholipid and secreted *agr*-regulated PSMs, which inhibited the phospholipid-daptomycin interaction [104].

Analysis of *de novo* mutations in >1000 *S *. *aureus* genomes from 105 infected patients with prior nasal colonization revealed that adaptive mutations in pathogenesis-associated genes including *agr* were enriched in infecting but not nasal-colonizing bacteria indicative of within host selection pressures [101]. Others have shown that events associated with *agr* inactivation result in *agr*-defective blood and nasal strain pairs that are enriched in mutations compared to pairs from wild-type controls [105]. These additional mutations outside the *agr* locus can contribute to diversification and adaptation during infection by *agr* mutants associated with poor patient outcomes [105].

In their analysis of >40 000 *S *. *aureus* genomes, Raghuram and co-workers [32] did not find any stable *agr*-defective strain lineages. This is consistent with previous suggestions that *agr* defective strains may be unable to establish and maintain circulating populations outside their original location [32, 99]. While the host factors responsible for driving the selection of *agr* dysfunctional mutants have not yet been identified, host resistance should not be overlooked as indicated by Thänert *et al*. [106] who compared susceptible (A/J) and resistant (C57BL/6) mouse strains with respect to *S. aureus* infection and virulence gene expression.

Strains with mutations in *agr* are capable of prolonged intracellular survival. Although they cannot escape phagosomes, they induce less cell death and so survive in greater numbers within host cells [107]. *agr* mutants can also persist via an alternative intracellular pathway in endothelial cells involving LC3^+^ vesicles [107]. Furthermore, sub-populations of slow-growing *S. aureus* small colony variants (SCVs) able to survive within host cells are frequently recovered from chronic and recurrent infections [108, 109]. These SCVs may be genetically stable with characteristic mutations in metabolic pathways or unstable and exhibiting increased expression of negative *agr* regulators (e.g. SigB, ArlRS and CodY). In both cases, a common characteristic of SCVs that arise from either altered electron transport or global regulatory pathway changes is reduced Agr activity.

These observations suggest that *S. aureus* populations progressing from colonization to infection at different body sites may be heterogenous with respect to *agr* expression, or consist of *agr*-functional cells or *agr*-defective mutants or mixed populations with the balance influencing the outcome. It is also apparent that *agr* mutant populations may contain a small fraction of phase variable cells capable of reverting to *agr*-functional cells [110]. This appears to arise at least *in vitro* via a mechanism involving a poly(A) tract alteration and a genetic duplication plus inversion event. This strategy has been suggested to act as cryptic insurance against host-mediated stress enabling the population to survive phagocytosis and sustain infection [110].

An explanation for why *S. aureus agr* mutants exhibit reduced virulence in animal models but are frequently isolated from clinical samples was suggested by Pollitt *et al*. [111] using a waxworm larvae infection model. They showed that *agr*-dependent QS is a beneficial social trait in which *agr* mutant ‘cheats’, which neither produce nor respond to AIPs exploit *agr* functional AIP-producing co-operators. However, while these data provide an explanation for mixed populations in toxigenic infections, they do not account for chronic, biofilm-associated clinical *S. aureus* infections where homogeneous *agr*-negative populations can rapidly emerge [112]. It should also be noted that *S. aureus* isolates exhibiting low levels of exotoxins are not necessarily *agr* mutants highlighting the likely existence of other, novel exotoxin regulators [113].

### *agr* dysfunction – the molecular basis

DNA sequence analysis of *agr*-dysfunctional *S. aureus* clinical isolates has revealed frameshifts, insertions, deletions and substitutions in the *agrBDCA* operon [87, 97, 98, 113, 114]. In their analysis of over 40 000 *S *. *aureus* genomes Raghuram *et al*. [32] found that >5 % had *agr* operon frameshifts. These were almost exclusively found in *agrA* and *agrC* with the latter having accumulated the greatest number of different mutations. The insertion of an extra adenine at the 3′ end of *agrA* was the most common frameshift and is known to result in delayed *agr* activation and haemolysin production [115]. Interestingly, when comparing the *agr* histidine kinase (*agrC*) and response regulator (*agrA*) genes with other *S. aureus* TCSs (*arl, kdp*, *nre*, *pho*, *srr* and *wal*), the frequency of mutations in *agr* is highly enriched [32]. The frequent occurrence of common but independently acquired convergent mutations may be an adaptive response to specific host selective pressures.

Some of these naturally occurring changes have either been predicted or experimentally demonstrated to result in the complete loss of *agr* functionality. For example, Mairpady Shambat *et al*. [116] isolated an MRSA strain harbouring a single AgrC Y223C cytoplasmic domain substitution that switched the virulence phenotype from cytotoxic to colonizing that could be reversed by mutating back to C223Y. However, not all naturally occurring *agr* mutations are likely to be inactivating but may instead modify the timing and/or the strength of *agr* induction. Sloan *et al*. [87] identified a number of natural mutations associated with reduced cytotoxicity also linked to the cytoplasmic domain of AgrC. These delayed the onset and accumulation of AIPs and exhibited impaired AgrC-AIP responsiveness as revealed by the increased threshold for AgrC activation. Exotoxin production, in this case Panton–Valentin leucocidin production, could be restored by exogenous provision of the cognate AIP at the time of inoculation indicating that delayed activation of *agr* autoinduction and consequently failure to express RNAIII results in the lack of exotoxins. Molecular dynamics simulations from *in silico* engineered point mutations in the AgrC cytoplasmic domain revealed subtle changes that alter both domain conformation and relative domain orientation [87]. The efficiency of the rheostat-like behaviour of AgrC in which AIP binding induces the twisting of a helical linker relative to the cytoplasmic domain and subsequent dimerization [25] is likely to be impaired by these mutations. Consequently, a greater magnitude of ‘input’ into the rheostat-like mechanism will be required in order to produce the same response. In turn, this would impact on the efficiency of the *agr* autoinduction circuitry. Such conformational rearrangements of key functional subdomains in these AgrC cytoplasmic domain mutants highlight the cooperative responses of protein structures involving dimerization, ATP binding and phosphorylation, as well as sites involved in AgrA interactions. Whether increasing the threshold for *agr* activation offers a fitness advantage remains to be established.

### Intra- and inter-species *agr*-mediated competitive interference among the Staphylococci

In *S. aureus,* strains belonging to one *agr* group produce an AIP that cross-inhibits each of the other three *agr* groups, giving rise to the concept of ‘competitive interference’ [27, 117]. In laboratory experiments in broth inoculated with mixed cultures, the *agr* group did not impact on competitiveness [117]. This is perhaps not surprising given that such interference is at the level of *agr* expression rather than growth. *In vivo* in a *Manduca sexta* (tobacco hornworm) infection model, despite the genetic diversity of the *S. aureus* strains tested, differences in the fitness of competing strains belonging to different *agr* groups were observed [117].

There have been very few documented reports of clinical samples containing mixed *agr* group isolates. In one case a patient was reported to have an *S. aureus agr* group 1 blood isolate and a group 2 wound isolate [97] while in another, a CF sputum sample contained genome sequences from both *agr* group 1 and 2 strains [32]. A comparison of consecutive and co-colonizing strains in healthy individuals or those with CF revealed that strain replacement was accompanied by a change in the *agr* group in 63 and 80 % of the two cohorts, respectively [118]. Co-colonizing strains from CF belonged to interfering *agr* groups in six of ten cases whereas for healthy individuals, nasal co-colonization with strains belonging to different *agr* groups was rarely observed [118]. However, in CF sputum, where *agr* is not expressed [119], no cross-group inhibitory AIPs will be synthesized making *agr* group competition unlikely.

Competitive interference however does occur during interactions between *S. aureus* and the CoNS, which produce a broad range of AIPs [37]. They constitute a diverse group of at least 38 different staphylococcal species that are primarily found on the healthy skin and mucous membranes of humans and other mammals, alongside other bacterial genera including corynebacteria, streptococci and micrococci [120, 121]. In a screen of culture supernatants prepared from 52 staphylococcal isolates representing 17 different species obtained from dogs, cows, horses, mink, cats, pigs and birds, Canovas *et al*. [21] identified 17 different species that inhibited *S. aureus agr*. Of 54 CoNS isolates obtained from 25 pig nasal swabs, the eight different species capable of inhibiting *S. aureus agr* included *Staphylococcus hyicus, Staphylococcus simulans, Staphylococcus arlettae, Staphylococcus lentus* and *Staphylococcus chromogenes* [2]. However, *Staphylococcus sciuri* and *Staphylococcus xylosus* were unable to inhibit *S. aureus agr* [2]. The presence of specific CoNS species in pig nares has been associated with reduced MRSA colonization and of relevance to intensive pig farming where the transmission of livestock associated MRSA from pigs to humans is a potential health risk [2].

The most common CoNS skin species are *S. epidermidis *, *S. hominis *, *Staphylococcus haemolyticus *, *Staphylococcus capitis *, *Staphylococcus lugdunensis* and *Staphylococcus warneri *. Most CoNS are relatively harmless, beneficial commensals that actively contribute to shaping the skin microbiota and the cutaneous immune response, promoting tissue-repair and combatting the external threat posed by invading pathogens such as *S. aureus* and group A streptococci [1, 122–124]. However, certain CoNS species also have dual lifestyles as colonizers and opportunistic pathogens. *S. epidermidis,* regarded as a keystone skin commensal, is frequently responsible for blood stream infections associated with implanted medical devices including intravascular catheters, prosthetic vascular grafts and heart valves, cardiac devices and coronary stents [121]. Considering their genetic flexibility and integrity of the skin barrier, it has been proposed that the beneficial or harmful behaviour of *S. epidermidis* may vary depending on the specific strain and interactive context [123, 124]. Indeed, analysis of 1482 *S *. *epidermidis* genomes from the skin of five healthy individuals established that they had evolved from multiple founder lineages rather than a single colonizer [125]. *S. saprophyticus *, a ‘moderately’ pathogenic CoNS species found in the genito-urinary tract is the second most frequent cause of uncomplicated lower urinary tract infection in young women [121].

Among CoNS species, the *agrBDCA* genes are widespread but highly divergent and offer opportunities for competitive interference within and between species [126]. Given the diversity of CoNS colonizing the same environmental niche, there is considerable scope for competitive interference at both intra- and inter-species levels, promoting colonization and suppressing virulence factor production by both *S. aureus* and CoNS capable of adopting pathogenic lifestyles [1, 37]. Such control will ultimately depend not only on competitive *agr* interference but also on the production of antimicrobials by CoNS such as PSMs that selectively inhibit *S. aureus* and which may themselves be *agr*-regulated [122].

Based on their AgrD peptide sequences, most CoNS, in common with *S. aureus* can be divided into different *agr* subgroups; between two and six depending on the species (Table 1). In *S. hominis* there are six *agr* groups [127, 128] while of the four *S. epidermidis agr* groups, healthy human skin is commonly dominated by a large proportion of *S. epidermidis agr* group I strains together with smaller sub-populations of *agr*-II and -III strains [125, 129]. To date, CoNS species where chemical structures have been confirmed, produce AIPs that, in common with *S. aureus *, are usually between seven and nine amino acid residues and incorporate a thiolactone ring. The exceptions are *S. intermedius* that produces a lactone [130] and *S. epidermidis* which makes three AIPs with extended seven amino acid residue exocyclic tails [125, 129]. The *S. intermedius* AIP lactone is a functional autoinducer in which the switch from Cys to Ser may have arisen via spontaneous point mutation [130]. Neither AgrB1 nor AgrB2 from *S. aureus* could enzymatically process a Ser containing AgrD pro-peptide to generate the mature AIP nor could the *S. aureus* AIP-1 or AIP-2 lactones activate the *S. aureus* AgrC1 or AgrC2 receptors [28, 130]. Interestingly the readily *S*-oxidized C-terminal Met present in *S. aureus* AIP-1 and AIP-4 is very rarely found in other staphylococci except for *S. argenteus* and *S. schweitzeri *, which, respectively, make the same AIPs as *S. aureus agr* groups I and IV [37].

**Table 1.** Competitive interference between AIPs produced by CoNS and the *agr* groups of *S. aureus* and *S. epidermidis
*

Coagulase-negative staphylococci			* S. aureus *				* S. epidermidis *		
**Species**	**Autoinducing peptide**		***agrI***	***agrII***	***agrIII***	***agrIV***	***agrI***	***agrII***	***agrIII***
*** S. epidermidis ***	**AIP-1**	**DSV-[CASYF]**	**166**	**>1000**	**13**	**>1000**	–	**9.64**	**34.3**
	**AIP-2**	**NASKYNP-[CSNYL]**	–	–	–	–	**13.9**	–	–
	**AIP-3**	**NAAKYNP-[CASYL]**	–	–	–	–	**2.13**		
*** S. caprae ***	**AIP**	**YST-[CSYYF]**	**0.6**	**0.26**	**0.2**	**9.0**	–	–	–
*** S. hominis ***	**AIP-1**	**SYNV-[CGGYF]**	**13**	**31**	**5**	**2910**	**NC**	**34**	**16**
	**AIP-2**	**SYSP-[CATYF]**	**15**	**2109**	**3**	**1130**	**20**	**19**	**62**
	**AIP-3**	**TYST-[CYGYF]**	**11**	**4**	**6**	**NC**	**4**	**3**	**3**
	**AIP-4**	**TINT-[CGGYF]**	**128**	**140**	**37**	**NC**	**237**	**93**	**28**
	**AIP-5**	**SQTV-CSGYF]**	**43**	**59**	**4**	**3809**	**10**	**22**	**2**
*** S. warneri ***	**AIP-1**	**YSP-[CTNFF]**	**10**	**4**	**13**	**146**	**3**	**19**	–
	**AIP-2**	**ANP-[CAMFY]**	**2**	**30**	**2**	**2**	–	–	–
*** S. simulans ***	**AIP-1**	**KYNP-[CLGFL]**	**2.2**	**1.1**	**3.5**	**23**	–	–	–
	**AIP-2**	**KYYP-[CWGYF]**	**1.6**	**1.5**	**11.5**	**40**	–	–	–
	**AIP-3**	**KYNP-[CWGYF]**	**1.7**	**6**	**3.2**	**48**	–	–	–
*** S. schleiferi ***	**AIP**	**KYPF-[CIGYF]**	**2.8**	**86**	**80**	–	–	–	–
*** S. lugdunensis ***	**AIP-1**	**DI-[CNGYF]**	**384**	**419**	**36.6**	**>1000**	–	–	–
*** S. hyicus ***	**AIP**	**KINP-[CTVFF]**	**3.3**	**350**	**4**	**180**	–	–	–
*** S. chromogenes ***	**AIP**	**SINP-[CTGFF]**	**15**	**200**	**60**	**350**	–	–	–
*** S. haemolyticus ***	**AIP**	**SFTP-[CTTYF]**	**340**	**IA**	**340**	**IA**	–	–	–
*** S. vitulinus ***	**AIP**	**VIRG-[CTAFL]**	**190**	**800**	**690**	**IA**	–	–	–

The square brackets denote amino acid residues within the AIP macrocycle. IC_50_ values compiled from [37, 128, 133–136]; NC, not calculated, likely to be a weak inhibitor; IA, not active at highest dose tested; – not available.

In general, there is relatively little information on the target genes regulated via *agr* in different CoNS species. Given the difficulty in genetically manipulating CoNS, Severn *et al*. [128] used the AgrA inhibitor apicidin to transcriptionally profile genes regulated by an *agr* group-I *S. hominis* commensal and identified ~40 down- and ~7 up-regulated genes. Down-regulated genes coding for PSMs, a predicted lipase, acetoin production and multiple transcriptional regulators as well as *agrB*, *agrD* and RNAIII were identified [128]. The up-regulated *S. hominis* genes were of unknown function but likely to be involved in metabolism and sensing. In *S. epidermidis *, one of the most abundant CoNS skin colonizers in which a functional *agr* system enhances skin colonization [129], genes down-regulated in an RNAIII mutant included protease and lipase exoenzymes, PSMs, δ-haemolysin, and *agr*. The haemolytic CoNS species *S. lugdunensis* can cause severe human infections but its repertoire of *agr*-dependent virulence determinants have not been well characterized. In a recent study, Chin *et al*. [131] showed that the *S. lugdunensis* synergistic haemolysins (SLUSH), the metalloprotease lugdulysin, a urease and a number of ABC transporters were downregulated in an *agr* mutant as were unidentified factors required to protect the organism from host innate immune defences.

Studies of AIP-mediated activation of CoNS *agr* systems or of competitive interference between CoNS species, or between CoNS and *S. aureus* are usually undertaken initially using spent culture supernatants and screened using *agr*P3 reporter gene fusions based on activation or inhibition of *S. aureus* or CoNS *agr* groups [2, 21, 128, 130]. While these highlight the diversity of CoNS capable of inhibiting one or more *S. aureus agr* groups, such data requires validation via synthesis of the predicted AIP. This then facilitates quantitative determination (EC_50_ or IC_50_) of the agonist or antagonist activities of a specific AIP. In experiments employing *S. epidermidis agr*P3-gfp reporter fusions and spent culture supernatants [129], interference between *S. epidermidis agr* group I and groups II and III but not between *agr* groups II and III were observed. These data were subsequently confirmed using the corresponding synthetic AIPs [129, 132, 133] (Table 1). In contrast to AIP-1, both AIP-2 and AIP-3 have extended exocyclic tails that likely account for the differential activity. By quantifying δ-haemolysin as a read-out for *agr* inhibition in different *S. aureus* strains, *S. epidermidis* AIP-1 was reported to inhibit *S. aureus agr* groups I to III but not group IV. Conversely, *S. aureus* AIP-4 was the only *S. aureus* AIP found to inhibit *S. epidermidis agr* group I [132]. Using an *agr*P3 reporter, Yang *et al*., [133] observed that *S. aureus* AIP-2 but not AIP-1, AIP-3 or AIP-4 inhibited *S. epidermidis agr* group I [133]. *S. hominis* AIP-1 to −6 all inhibited *S. aureus agr* groups I, II and III with IC_50_s ranging from 3 to 140 nM except for AIP-2 which had little activity against *S. aureus agr* group II [126] (Table 1). The *S. hominis* AIPs were all active against the three *S. epidermidis agr* groups except for AIP-1, which lacked antagonistic activity towards *S. epidermidis agr* group I [128]. The strongest *S. hominis* intraspecies interactions were observed between *agr* groups I and II, which were the two most common *S. hominis agr* types identified on the skin of a group of 14 human volunteers and in database genome sequences [125, 128]. In common with *S. epidermidis *, none of the *S. hominis* AIPs were effective antagonists of *S. aureus agr* group IV [128] although the IC_50_s for the *S. simulans* AIPs were in the 20–40 nM range [134] (Table 1) *S. simulans agr* types also displayed varying degrees of resistance to cross-inhibition from *S. aureus* AIPs, with *S. simulans agr* III being weakly susceptible to antagonism by *S. aureus* AIP-1 and AIP-4 [134]. Intraspecies interactions between *S. simulans agr* variants may also occur since *agr* group I is inhibited by AIPs-2 and 3 whereas *agr* groups II and III are not subject to cross-inhibition. Two potent *S. aureus* cross-group inhibitors are *S. warneri* AIP-2 and the *S. caprae* AIP [135] both of which are potent antagonists of all four *S. aureus agr* groups [136] (Table 1). Although inter-species *agr* activation is rarely observed, the AIPs of *Staphylococcus schleiferi* and *Staphylococcus hominis* (AIP-3) both induce *S. aureus agr* group IV [37, 128] (Table 1).

### Competitive interference and skin disease

*S. aureus* and especially CA-MRSA strains are the most common causes of skin and soft tissue infections in humans [1, 137, 138]. The severity of atopic dermatitis (AD), a chronic disease of unclear aetiology is characterized by dry, itchy and inflamed skin and by dysbiosis of the skin microbiota. *S. aureus* colonizes AD patients’ skin lesions and exacerbates disease by promoting inflammation and degradation of the skin barrier function. These have all been linked with *S. aureus* exotoxins, superantigens, exoproteases and PSMs [137] and also with an overabundance of *S. epidermidis* strains producing the cysteine protease EcpA [123, 139]. Metagenomic analysis of the AD skin microbiome revealed that an increase in the relative abundance of *S. aureus* in patients with active AD correlated with a lower CoNS AIPs to *S. aureus* ratio, thus reducing the ability of the CoNS to inhibit the *S. aureus agr* system [127]. In a further study involving 268 Japanese infants aged 1 to 6 months, 121 were colonized with *S. aureus* at 1 month irrespective of AD outcome [19]. However, colonization with *S. aureus* at 6 months increased the likelihood of developing AD. Selection for dysfunctional *agr* mutations primarily in *agrC* occurred in *S. aureus* strains from 6-month-old infants who did not develop AD. In an epicutaneous mouse inoculation model that induces *agr* and skin surface inflammation, the expression of a functional *S. aureus agr* system was found necessary for skin colonization and the development of AD-like inflammation [19]. There is therefore considerable interest in the therapeutic potential of commensal CoNS for suppressing *S. aureus* in AD either by production of potent *S. aureus* selective antimicrobials [122], via competitive interference [127] or both since antimicrobials such as the PSMs are regulated via *agr*.

The ability of a specific CONS species or the corresponding synthetic AIP to prevent or reduce skin colonization and damage by *S. aureus* CA-MRSA *agr* group I strain LAC has been extensively evaluated in murine epicutaneous (bacteria applied topically via gauze) and dermonecrosis (bacteria injected intradermally) skin models for *S. hominis* [127, 128], *S. caprae* [135], *S. simulans* [134] and *S. warneri* [136]. These *in vivo* investigations facilitated quantification of the dose-dependent efficacy of the CoNS AIPs or the corresponding CoNS strain with respect to reducing MRSA skin colonization, lesion morphologies and sizes, weight loss, bacterial burden and skin barrier integrity. *S. hominis* AIP-1 and AIP-2 both inhibited *S. aureus agr* group I activity on mouse skin and protected against epidermal damage by reducing skin lesion size, transepithelial water loss and inducing a productive host response without affecting *S. aureus* abundance [127, 128]. Co-challenge with *S. hominis* was not as effective as the *S. hominis* AIP-2 alone, which was suggested to be a consequence of lower levels of AIP-2 production on the skin by the human isolate employed or because mouse rather than human skin was used [128]. Similar results were obtained for MRSA co-infection with *S. simulans* [134], *S. warneri* [136] and *S. caprae* [135] and also in response to their respective AIPs. *S. warneri* AIP-2, which is more fivefold more potent *in vitro* than AIP-1, is one of the few naturally occurring AIPs that can inhibit *S. aureus agr* group IV [136]. The latter has been associated with scalded skin syndrome and the production of exfoliative toxins. In a dermonecrotic model, the *S. warneri* AIP-2 reduced skin lesion size and protected mice infected with an MRSA *agr* group IV strain from weight loss and skin damage [136]. Apart from *S. caprae,* none of the other CoNS AIPs reduced the MRSA *agr* group I bacterial burden during the course of infection to levels comparable to those observed for an MRSA *agr* deletion mutant [135]. This appears to be a consequence of the *S. caprae* AIP sensitizing MRSA to neutrophil-mediated clearance rather than a direct immunogenic effect of the AIP. Why this did not occur with the other CoNS AIPs is not clear, but may relate to the greater *in vivo* activity of the *S. caprae* AIP since significant protection was achieved at 10 µg per mouse [135] whereas comparable protection required 50 µg of the other CoNS AIPs [125, 128, 134, 136]. For each of the AIPs tested, significant protection was observed for a single AIP dose when administered at the time of infection although more subtle therapeutic effects were observed when AIPS were delivered later in the course of infection [127, 128, 134–136].

These findings provide the tantalizing prospect of employing CoNS or AIPs as a means of limiting skin infections caused by *S. aureus* especially in the context of multi-antibiotic resistant MRSA. Further work will need to consider asymptomatic mouse skin models, the differences between human and mouse microflora as well as innate immune and healing responses. Given that skin is colonized by diverse commensals producing many different AIPs and antimicrobials, their overall impact is likely to be complex [125]. As yet, there is little information on competitive interference when multiple CoNS strains and AIPs are involved or even other commensal bacteria such as the *Corynebacterium* species, which are capable of inhibiting *S. aureus agr* groups I to III via an unknown mechanism [140]. In a recent, double-blinded, randomized phase I clinical trial, the safety of an *S. hominis* strain delivered topically to forearm skin was evaluated over 7 days. Promisingly, the trial met its primary endpoint of safety with those treated with *S. hominis* experiencing fewer AD-associated adverse events and a reduction in *S. aureus* [141].

## Concluding remarks

The function and dysfunction via inhibition or mutation of the *agr* system play key roles in the adaptive behaviour and fitness of *S. aureus* in different host environments, in response to antibiotics and during encounters with other staphylococcal species especially on the skin. Considerable progress in understanding the molecular mechanisms underlying *agr-*dependent QS has been gained although there is still relatively little high-resolution information on the 3D structures of AgrC, AgrB and MroQ or on the nature of the respective AIP and AgrD substrate-binding sites on each of these transmembrane proteins. Whether MroQ processes its thiolactone substrate on the inner or outer face of the cytoplasmic membrane and how AIPs are exported remain to be elucidated. Given the diversity of AIPs leading to extensive competitive interference, it is perhaps surprising that there is little cross-activation within or between staphylococcal species. Whether AIP-driven interference occurs between staphylococci and other Gram-positive bacteria that harbour *agr* systems especially in complex microbial communities is not yet known. We also have little understanding of the selective pressures that led to the emergence of different *agr* groups or indeed why four *agr* groups evolved in *S. aureus* with no evidence of intermediates. Given the rise of multi-antibiotic-resistant MRSA, the *agr* system does offer multiple druggable molecular targets for inhibitors including AgrA, AgrB and AgrC [18]. However, despite the *in vitro* identification of highly active AIP analogues and non-peptidic *agr* inhibitors much more *in vivo* work will be required before any ‘hits’ with the appropriate pharmacological and pharmacokinetic properties can enter human clinical trials. These are likely to be complicated by the association between *agr* dysfunction, biofilm formation and chronic infections. Nevertheless, employing CoNS strains or antagonistic AIPs as a means of limiting *S. aureus* skin infections appears to offer a realistic therapeutic opportunity [141].
